# Patterns in reef fish assemblages: Insights from the Chagos Archipelago

**DOI:** 10.1371/journal.pone.0191448

**Published:** 2018-01-19

**Authors:** Melita Samoilys, Ronan Roche, Heather Koldewey, John Turner

**Affiliations:** 1 CORDIO East Africa, Mombasa, Kenya; 2 Zoology Department, University of Oxford, Oxford, United Kingdom; 3 School of Ocean Sciences, Bangor University, Bangor, United Kingdom; 4 Conservation Programmes, Zoological Society of London, London, United Kingdom; 5 Centre for Ecology & Conservation, University of Exeter Cornwall Campus, Penryn, Cornwall, United Kingdom; Department of Agriculture and Water Resources, AUSTRALIA

## Abstract

Understanding the drivers of variability in the composition of fish assemblages across the Indo-Pacific region is crucial to support coral reef ecosystem resilience. Whilst numerous relationships and feedback mechanisms between the functional roles of coral reef fishes and reef benthic composition have been investigated, certain key groups, such as the herbivores, are widely suggested to maintain reefs in a coral-dominated state. Examining links between fishes and reef benthos is complicated by the interactions between natural processes, disturbance events and anthropogenic impacts, particularly fishing pressure. This study examined fish assemblages and associated benthic variables across five atolls within the Chagos Archipelago, where fishing pressure is largely absent, to better understand these relationships. We found high variability in fish assemblages among atolls and sites across the archipelago, especially for key groups such as a suite of grazer-detritivore surgeonfish, and the parrotfishes which varied in density over 40-fold between sites. Differences in fish assemblages were significantly associated with variable levels of both live and recently dead coral cover and rugosity. We suggest these results reflect differing coral recovery trajectories following coral bleaching events and a strong influence of ‘bottom-up’ control mechanisms on fish assemblages. Species level analyses revealed that *Scarus niger*, *Acanthurus nigrofuscus* and *Chlorurus strongylocephalos* were key species driving differences in fish assemblage structure. Clarifying the trophic roles of herbivorous and detritivorous reef fishes will require species-level studies, which also examine feeding behaviour, to fully understand their contribution in maintaining reef resilience to climate change and fishing impacts.

## Introduction

Coral reefs are complex and highly biodiverse systems that are subject to a broad range of natural and anthropogenic factors, operating from local to global scales, which drive or impact reef fish population abundance and assemblage structure [1–4]. Reef degradation from fishing pressure and climate-change induced coral bleaching and mortality have been invoked to explain patterns in the structure of coral reef fish assemblages across multiple scales in the Indo-Pacific [5–7]. Other studies point to scale dependence in drivers of fish assemblages with geomorphology and biogeography, for example, playing a significant role at larger regional scales, and fishing and reef benthic structure operating at local scales [8–10]. Understanding the mechanisms by which these drivers interact and their relative contributions to controlling reef fish assemblages is critical in underpinning conservation planning and effective reef fisheries management.

One of the dominant paradigms used to explain impacts from the external stressors of climate change and fishing on coral reefs and their fish assemblages revolves around potential shifts from coral to algal-dominated reef states [11,12]. Herbivorous fishes have been shown to play a leading role in preventing this shift by controlling algal abundance [2,13]. The regulatory pathways involve both resource (bottom-up) and predation (top-down) control of the reef ecosystem. Changes in coral cover represent bottom-up control while top-down control is seen when herbivores are depleted through fishing activities, which can lead to their functional role becoming compromised [4,14]. Coral reef fish assemblages are known to vary in relation to several environmental characteristics such as exposure to oceanic conditions, rugosity, depth, benthic composition and recent coral mortality [8–10,15–18]. Bottom-up control of reef fish populations by reef benthic composition has been well established [10,15,18–20], and long-term studies in the Philippines, for example, have shown that this pathway is the primary driver of the herbivorous parrotfishes [21]. Thus, top-down and bottom-up pathways can either dominate or co-occur, depending on the characteristics within the coral reef ecosystem.

From a management perspective, it is important to be able to attribute the relative contribution of casual factors driving the structure of reef fish assemblages. The objective of this study was to determine which of a range of largely biotic factors may be driving the structure of reef fish assemblages in the absence of fishing. Our hypothesis was that without the top-down influence of fishing in the Chagos Archipelago the fish assemblages should reflect the relative contribution of natural drivers, both bottom up (e.g. food availability) and top-down (e.g. predation), of fish populations, and one anthropogenic stressor—coral mortality related to bleaching events. We also sought to describe the characteristic reef fish assemblages of the atolls of the Chagos Archipelago to build on earlier work that examined fish responses to declines in coral cover caused by the coral bleaching event of 1998 [22] and found little change in reef fish species richness except in corallivores [23]. We also examined the abundance and biomass of reef fishes from the full range of trophic groups to test for relationships between trophic group and reef benthic composition and so examine the functional roles of fish species in reef resilience.

The Chagos Archipelago (British Indian Ocean Territory) is an isolated archipelago of atolls spanning ~60,000 km^2^ and 2 degrees of latitude on the north-eastern border of the western Indian Ocean Province [24–26], with an area of ~9,400 km^2^ of shallow coral reefs (<40m depth) [27]. The islands are uninhabited except for the southern-most atoll, Diego Garcia, which is classified as a Permanent Joint Operating Base of the UK and US governments and hosts a US naval support facility. The archipelago, with the exception of Diego Garcia where a recreational fishery is permitted, was declared a no-take marine protected area (MPA) in 2010 by the UK Government [26]. Indeed, reef fish biomass in the Chagos Archipelago is demonstrably one of the highest of any coral reef ecosystem in the Indo-Pacific [23]. The Chagos Archipelago therefore provides an ideal location for investigating the relationship between fish assemblages and variability in reef benthic habitat and typology, in the absence of impacts from fishing and human populations. Our study assumed that reef fish species distributions did not differ biogeographically across the Chagos Archipelago due to the direction of major current systems in the western Indian Ocean (WIO), and the connectivity of the pelagic larvae of most reef fish [28–30]. We do, however, recognise that self-recruitment [31] and local oceanographic dynamics [32] within and among atolls of the archipelago may affect larval recruitment patterns. An earlier study reported that reef fish assemblages were highly homogeneous across the northern atolls [33]. Here we use datasets from a range of atolls in the archipelago, from the northernmost atolls to Diego Garcia in the south, to examine variation in the abundance and species structure of fish assemblages, and to identify drivers of this variability.

By confining this study to an isolated archipelago of reefs that are relatively unfished and free of pollution and development, this study contributes to a better understanding of intact Indian Ocean reef fish assemblages. As such, it provides a regional context for interpreting coral reef fish assemblages in the wider Indian Ocean where anthropogenic impacts are more prevalent.

## Methods

### Study sites

**Fig 1:**
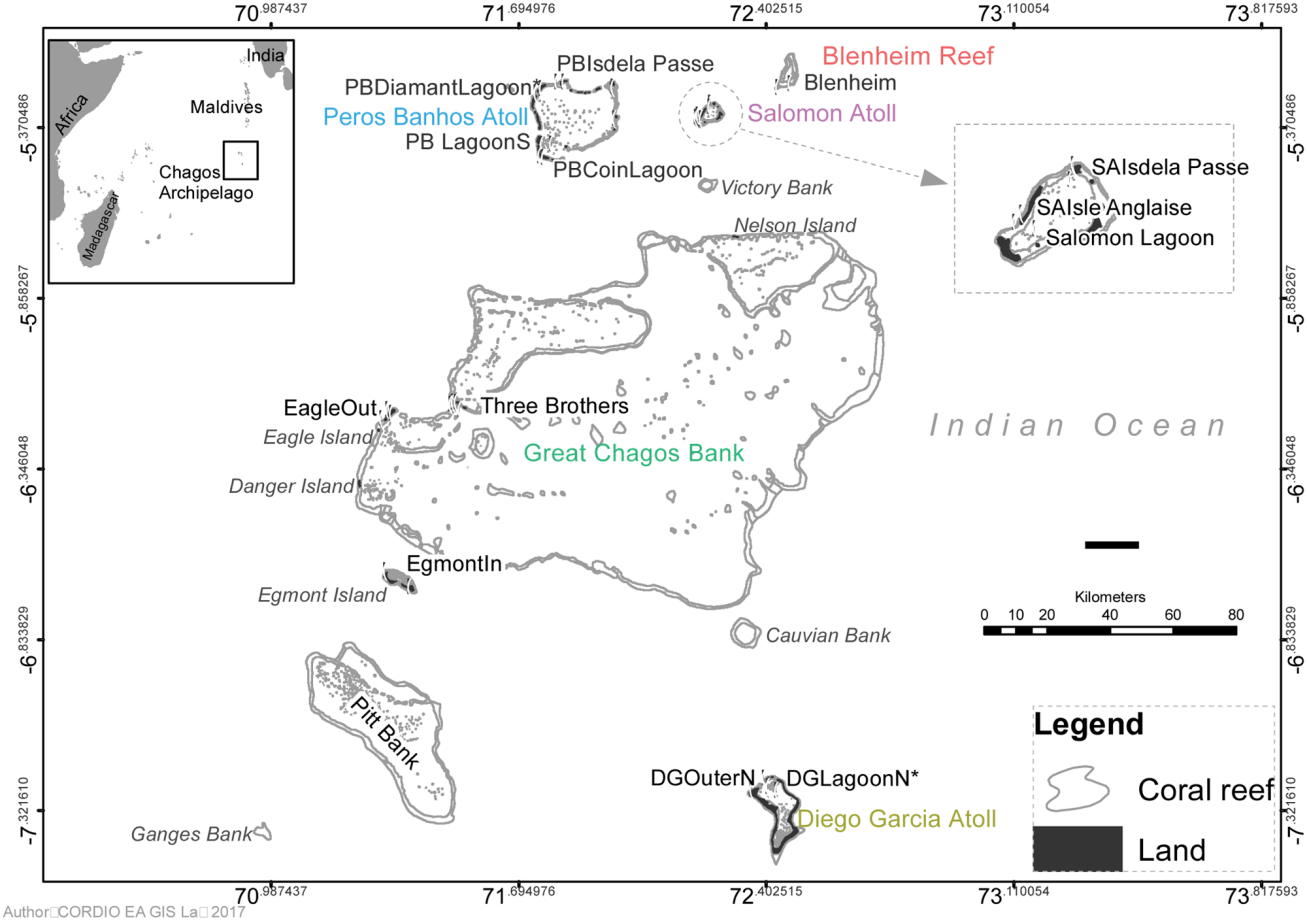
Map of the Chagos Archipelago showing atolls surveyed and locations of dive survey sites.

We surveyed reef fish assemblages and coral reef benthic assemblages in March 2014 at a total of 13 (fish) and 11 (benthic) sites across 5 atolls in the Chagos Archipelago (decimal minutes: 05.237333 S 71.81498 E to 07.26195 S 72.44333 E, Fig 1, S1 Table). Locations included the fully submerged Blenheim Reef atoll, reefs fringing islands on the west side of the Great Chagos Bank (GCB) and the large, well formed Peros Banhos and Salomon atolls. Reef types were defined based on the Andrefoute et al. [34] classification of coral reefs and included forereefs and terraces on the outside of the atolls and pinnacles and inner slopes in the atoll lagoons (S1 Table). These were categorised as exposed (outside atolls) or protected (inside lagoons) from oceanic seas. The British Indian Ocean Territory Administration Section of the Foreign and Commonwealth Office, UK Government, granted the research permit to the Darwin Initiative 2014 Expedition to work within the whole Territory. Permission was granted to all authors to visit and dive in the strict nature reserves of the Chagos Archipelago Marine Park.

### Benthic surveys

Underwater video transects were recorded using a Sony HDRCX550 camera in a Light and Motion Bluefin housing with Fathom 90 wide angle port and red filter, onto which red lasers with a spacing of 10 cm were mounted to provide scale. Surveys were conducted at each site which ranged in depth from 5–25m. The video aimed for a constant speed (~0.1 m s^-1^), with a 10 min transect within each of four depth zones (25–20 m, 20–15 m, 15–10 m, 10–5 m) approximately 1 m above the substrate [35]. Percentage cover of all hard coral and *Acropora* spp. alone, dead coral (defined as recently dead coral skeleton with intact corallite structure), soft coral, crustose coralline algae (CCA), fleshy macroalgae, turf algae, calcareous substrate, sand/rubble and all other benthos were assessed by randomly selecting 20 video frames from each depth range, and recording what lay beneath 15 randomly selected points per frame, for a total of 300 points per transect (thus 1,200 points per site), assigned using Coral Point Count software [36]. The rugosity of the reef along each transect was estimated visually using a six point scale following Polunin and Roberts [37], ranging from no vertical structural complexity to highly-developed reefs with large coral colonies, caves and crevasses.

### Fish surveys

All fish species from 13 pre-selected families that span the full range of trophic groups, from piscivores to detritivores (see S2 Table) were counted in 50 x 5 m transects. Two dives were conducted at each site which spanned approximately 300 m along the reef edge. In each dive 2–3 transects were run parallel to the reef edge (5–6 replicate transects in total per site). Transects were placed randomly at different depths to span the depth range of the reef, but a maximum dive depth of 26m was imposed by dive safety regulations. Fish counts at each site therefore had relatively broad depth ranges, depending on the reef profile (S1 Table). This design was used to maximise survey coverage of the fish assemblage on the reef. The fish survey sites corresponded to the dive sites at which the benthic video transects were placed; both methods spanned the same depth range at each site. Siganids (rabbitfishes) were not observed at all and therefore a total of 12 families were counted (S2 Table). The density and size classes of species were estimated using standard underwater visual census (UVC) techniques for coral reef fishes [38,39]. The size of all species >5 cm total length (TL) were estimated in 5 cm size classes (e.g. 6–10 cm TL, 11–15 cm TL, 16–20 cm TL etc), to obtain biomass values based on published length—weight relationships [40–42]. Biomass was calculated as a derived variable for the fish assemblage because it is a good indicator of energy flow within the coral reef ecosystem. A fixed size category for the smallest species was used because: simplifying counting procedures across a wide range of species improves accuracy [39,43]; any differences in biomass in these small species between sites will be smaller than the 5 cm size class accuracy used; and to enable these small species to be included in total biomass calculations. Fixed size classes were as follows: i) all Chaetontidae species were assigned a length size class of 6–10 cm, with the exception of *C*. *xanthocephalos*, *C*. *lineolatus* and *Heniochus* spp. which were recorded as 11–15 cm; ii) small acanthurids, *Ctenochaetus* spp., *Acanthurus nigrofuscus*, *A*. *leucosternon* and *Zebrasoma scopas*, were assigned a length size class of 11–15 cm; iii) *Centropyge* spp. (Pomacanthidae)–were assigned a length size class of 6–10 cm. A total of 110 species were identified and assigned to 12 functional trophic groups (piscivores, omnivores, corallivores, invertivores, planktivores, detritivores, grazer-detritivores and 5 herbivore categories, *sensu* Green and Bellwood [44]) using a classification system for the WIO [45] (S2 Table).

### Data analyses

For analyses, the data were organised into a series of matrices: i) fish species numerical density and biomass (13 sites); ii) fish functional group numerical density and biomass (13 sites); iii) benthic habitat variables (11 sites) that were natural log-transformed and standardised (11 variables).

#### Fish assemblages

Spatial autocorrelation in fish assemblages across the Chagos Archipelago was tested by implementing a Mantel test using the ade4 package [46] in R [47] on a matrix of geographic distances between sampling sites and a dissimilarity matrix based on fish density computed using the Bray-Curtis index. The Mantel statistic was further calculated within Peros Banhos, GCB and Salomon atolls, to test for a relationship with geographic distance between sites within atolls. Correlations between both numerical density and biomass matrices were tested for significance using 9999 permutations.

In order to visualise variation in the composition of fish assemblages across the archipelago, we used non-metric multidimensional scaling (nMDS) on Bray-Curtis dissimilarity distance measures obtained from fish data matrices of both abundance and biomass. To determine which of the fish trophic groups were significantly related to the ordination, we carried out random permutation testing using 9999 permutations. To further examine for groupings within the fish assemblage data, a Ward cluster analysis based on Euclidean distances was performed on hellinger-transformed data, using similarity profile analysis (SIMPROF) to test the significance of clustered groups [48].

#### Relationships between datasets

We tested for co-linearity within benthic variables and identified variables that were correlated at *r* ≥ 0.7. Three variables (calcareous substrate, sand/rubble, and other benthic) were removed from further analysis and no remaining pairwise correlations between variables greater than *r* = 0.53 were found. The remaining 8 variables were further tested by a variance inflation factor (VIF) analysis which found that each of the retained environmental variables resulted in a VIF of <10.

The Adonis function within the Vegan package [49] was used to examine for significant relationships between categorical variables (atoll, reef type and exposure) and the fish assemblages surveyed, also using permutation testing set at 9999 permutations. We used the envfit function within the Vegan package to estimate the direction and strengths of the correlation between the nMDS of fish species and the reef benthic variables surveyed.

Finally, we used a variation of the BIO-ENV [50] routine, termed BIO-BIO, to identify the subset of fish species which best correlated to the overall biological pattern of the dissimilarity matrix, using both numerical density and biomass data. They produced similar results, thus density alone was presented.

## Results

A total of 110 fish species from the 12 families were recorded across the Chagos Archipelago. The matrices of mean species density and biomass are provided in S3 and S4 Tables, respectively. Multivariate ANOVA (Adonis) permutation results found significant differences in the fish species matrices between atolls for both density and biomass datasets (F_4,12_ = 2.068, *P* = 0.002; F_4,12_ = 1.760, *P* = 0.010) and between three reef types (forereef; terrace & forereef; lagoons (2 types combined), S1 Table) for fish biomass (F_2,12_ = 1.673, *P* = 0.035). With a limited number of sites, these differences between reef types could not be tested further. There were no significant differences found in species’ density or biomass between sites classified as exposed (outer reefs) or protected (lagoon) sites (*P*>0.05).

Mantel tests indicated that dissimilarity in the fish assemblages using species density data was strongly related to geographic distance across the archipelago (Monte Carlo observation = 0.512; *P* = 0.002). However, within Peros Banhos, Salomon and GCB atolls there was no significant relationship between geographic distance between sites and the fish assemblages present (Peros Banhos: Monte Carlo Observation = -0.317, *P* = 0.499; Salomon: Monte Carlo Observation = -0.718, *P* = 0.835; GCB, Monte Carlo Observation = -0.224, *P* = 0.497).

**Fig 2:**
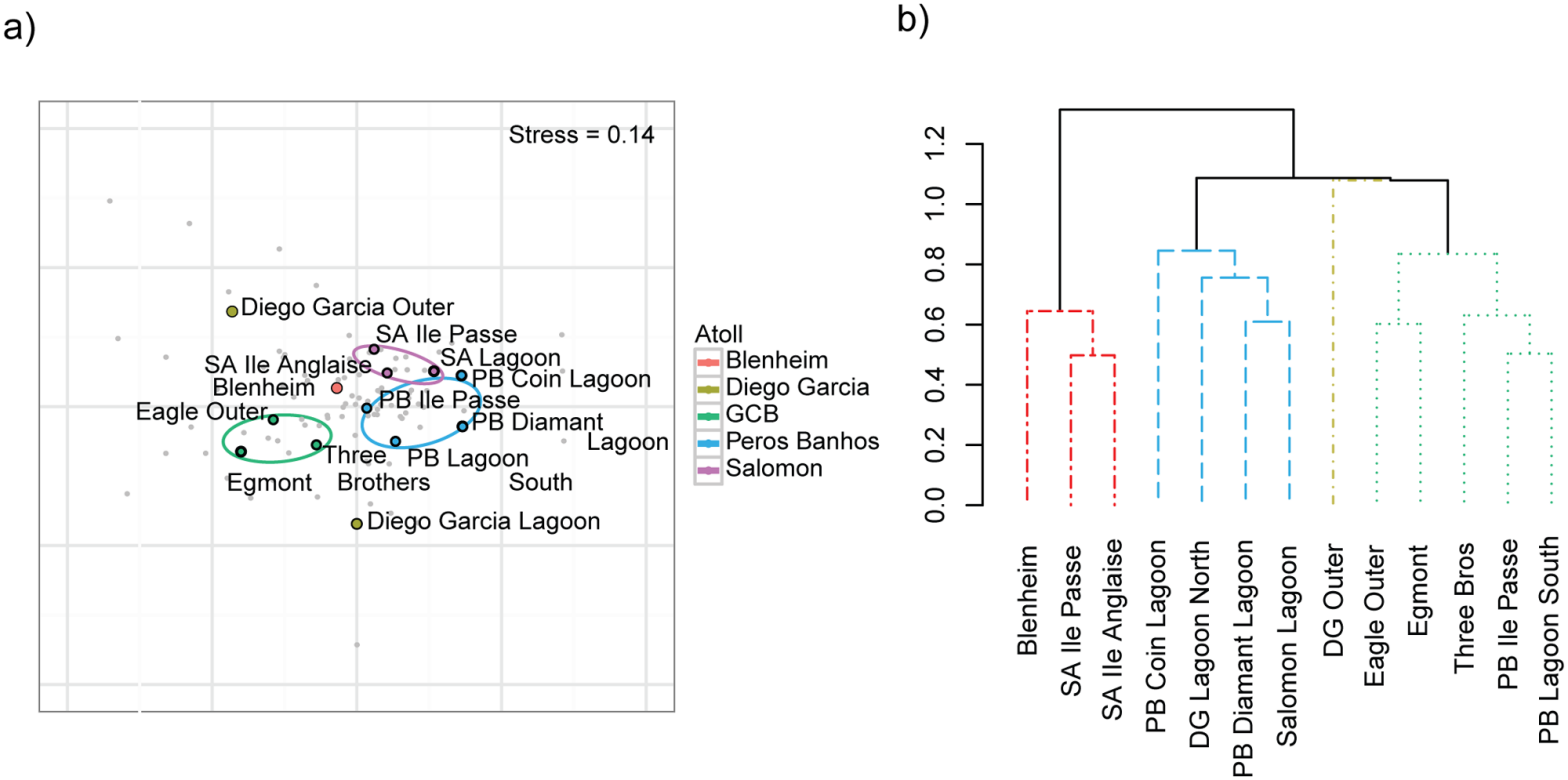
Spatial variation in reef fish species assemblages across the 13 sites in the Chagos Archipelago: a) non-metric multidimensional scaling plot; coloured ellipses show 95% confidence intervals of site grouping; b) Ward cluster analysis; colours in dendrogram highlight the four significantly different groups found (<0.6 dissimilarity).

**Fig 3:**
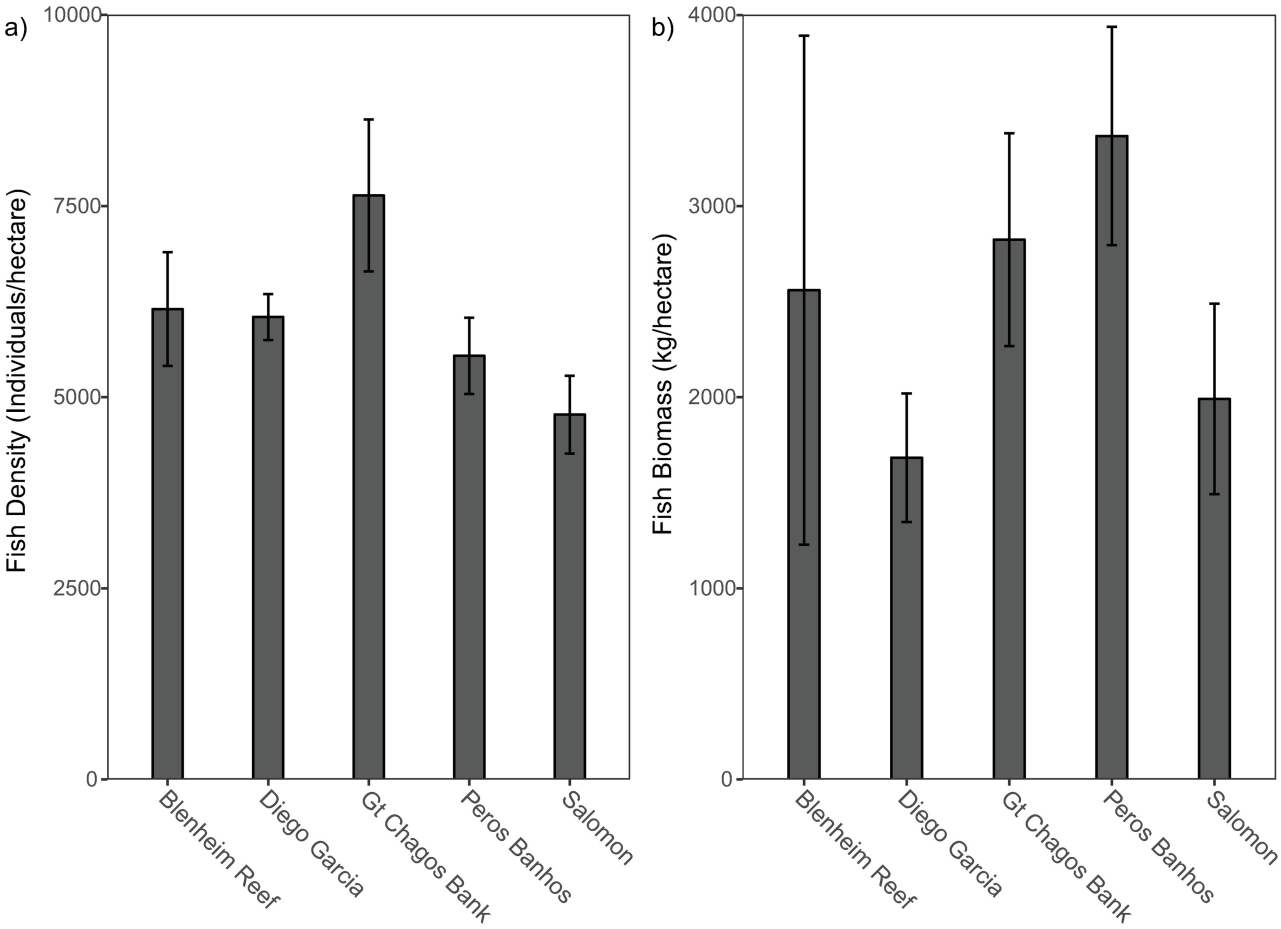
Total fish a) density (number of individuals per hectare) and b) biomass (kg per hectare) by atoll, based on 12 reef-associated families surveyed at 13 sites. Error bars are standard errors.

Ordination of species density data across the archipelago revealed three dissimilar groups corresponding to the atolls of Peros Banhos, Salomon and reefs of the GCB (Fig 2a). Fish assemblages at GCB separated most strongly from other atolls, while Peros Banhos and Salomon were more similar. These differences in fish assemblages were further verified by the Ward cluster analysis (Fig 2b), which showed four significant clusters (>60% dissimilarity) though one cluster (cluster 3) comprised of a single site—Diego Garcia Atoll’s terrace and forereef, which differed from all other sites (>1.0 dissimilarity). This Euclidian analysis provides a more detailed examination of dissimilarity in the fish assemblages across sites: cluster 1 was most dissimilar from all other sites and consisted of northern sites at Blenheim and Salomon Atoll forereefs; cluster 2 contained all lagoon sites, 3 from Peros Banhos but also 1 site from each of Salomon and Diego Garcia; whilst cluster 4 consisted of two sub-groups, Eagle and Egmont forereefs at GCB and Three Brothers forereef (GCB) and two Peros Banhos sites (a forereef and a lagoon pinnacle). Total fish density and biomass also showed broad-scale differences across the archipelago with the highest densities recorded on reefs at GCB, the highest biomass recorded at Peros Banhos Atoll and the lowest biomass at Diego Garcia Atoll (Fig 3).

**Fig 4:**
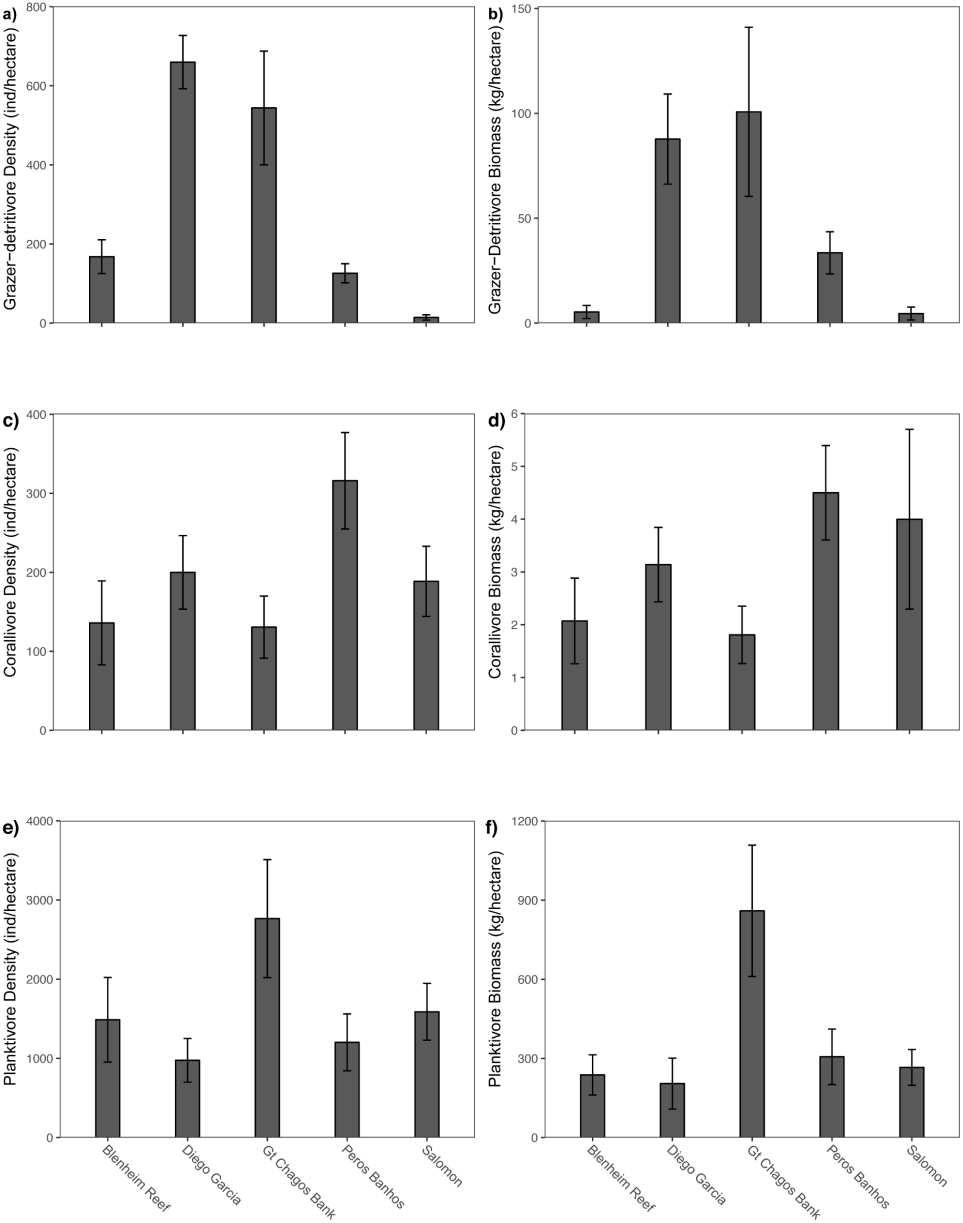
Mean density (number of individuals per hectare) and biomass kg per hectare) by atoll for the three functional trophic groups that were significantly related to fish assemblage differences. Error bars are standard errors. Functional trophic groups are explained in S2 Table.

When fish species were categorised into the 12 trophic groups, permutation tests showed only 3 trophic groups were significant in explaining the pattern in the species assemblages: grazer-detritivores and corallivores for fish density and grazer-detritivores and planktivores for fish biomass (Table 1, Fig 4). These three trophic groups all significantly explained fish density differences when the permutation test was stratified by atoll (Table 1). Grazer-detritivores comprise a group of acanthurids and the angelfishes *Centropye* spp. (S2 Table). Acanthurid species in this trophic group, such as *Acanthurus tennenti* and *A*. *xanthopterus*, typically feed on sand and hard surfaces to extract detritus and microbes, as well as epilithic algae. The densities and biomass of these grazer-detritivores were nearly three times greater at GCB and Diego Garcia compared to the other atolls (Fig 4), representing the largest difference in the fish assemblages across the archipelago. The corallivores consisted of six obligate coral feeding butterflyfishes out of the 18 Chaetodontidae observed in the Chagos Archipelago and were more abundant at Peros Banhos and Salomon atolls, compared to other reefs (Fig 4). When biomass was considered, the planktivores, comprised of balistid, acanthurid and chaetodontid species, differed significantly between the atolls with biomass at GCB three times higher than any of the other reef sites (Table 1, Fig 4).

**Table 1:** Random permutation results of 12 fish trophic groups showing only those significantly related to differences: a) across all sites and; b) stratified by atoll.

Density			Biomass		
*a) All sites*					
Trophic group	r^2^	*p*-value	Trophic group	r^2^	*p*-value
Grazer-detritivores	0.769	<0.001	Grazer-detritivores	0.792	<0.001
Corallivores	0.598	0.009	Planktivores	0.515	0.026
*b) Stratified by atoll*					
Grazer-detritivores	0.769	0.006	Grazer-detritivores	0.641	0.016
Planktivores	0.268	0.030	Planktivores	0.515	0.034
Corallivores	0.598	0.048			

### Benthic reef characteristics and fish assemblages

**Fig 5:**
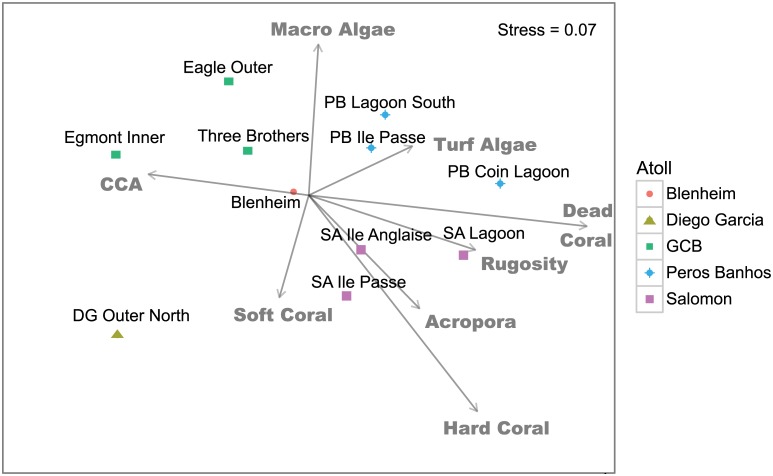
nMDS diagram showing the relationship between benthic variables at 11 reef sites overlaid on the fish assemblage ordination (see Fig 2) across the Chagos Archipelago. The relative contribution of each benthic variable is displayed by the length of the vector.

The benthic cover at reef sites was highly variable among the atolls of the archipelago (S5 Table). Total live coral cover ranged from 15.7% (±1.6 SD) to 47.2% (±24.1 SD), *Acropora* spp. coral cover from 1.1 (±1.4 SD), to 28.1% (±12.4 SD), and dead standing coral from 5.9% (±3.1 SD) to 26.4% (±13.1 SD). Non-metric multi-dimensional scaling of the relative contribution of the eight benthic variables to the differences between fish assemblages across the archipelago showed that reef sites grouped along two main axes (Fig 5): the Y axis with high macro-algae such as GCB reefs, versus sites with higher soft coral (Diego Garcia); and the X axis with sites with high hard coral, dead coral, live *Acropora*, rugosity and turf algae, at Salomon Atoll and Perhos Banhos, versus reefs at GCB with higher CCA. GCB reefs had the lowest levels of hard coral, ranging from 15.7% (±5.6 SD) to 28.7% (±17.7 SD). However, hard coral and dead coral (i.e. structural components) were the only benthic categories that were significantly related to differences in fish assemblage structure when analysed with fish density data; when tested with fish biomass data, rugosity also became significant (Table 2). When the permutation analysis was stratified by atoll, hard coral and dead coral were no longer significant; instead soft coral showed a significant correlation with fish density and CCA with fish biomass (Table 2). These results corroborate the geographic differences in fish assemblages between different atolls, driven by hard and dead coral cover, whereas within atolls only CCA and soft coral were significantly correlated with the fish species data matrices.

**Table 2:** Significant permutation correlations between benthos and the fish species matrix, for density and biomass at a) all sites and b) stratified by atoll.

Density			Biomass		
*a) All sites*					
Benthic Group	r^2^	*p*-value	Benthic group	r^2^	*p*-value
Hard Coral	0.63	0.021	Hard Coral	0.7	0.001
Dead Coral	0.66	0.013	Dead Coral	0.7	0.001
			Rugosity	0.55	0.034
*b) Stratified by atoll*					
Soft Coral	0.38	0.004	Crustose Corraline Algae	0.310	0.042

### Fish species

**Fig 6:**
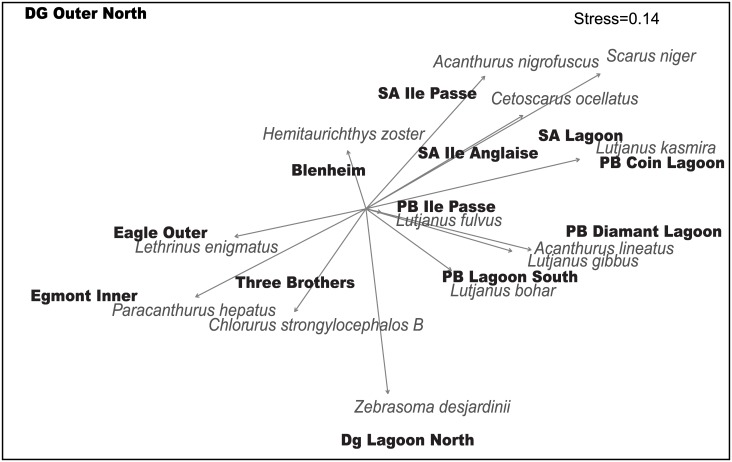
The relationship between individual species and the fish species density ordination based on the 13 fish survey sites.

A species-level ordination (BIOBIO) of the density of the 110 fish species which determined which species were most correlated with differences in the fish assemblages across all reef sites showed that 13 species best explained (rho = 0.832) the fish assemblages across the sites: *Acanthurus lineatus*, *A*. *nigrofuscus*, *Zebrasoma desjardinii* (grazers), *Cetoscarus ocellatus*, *Chlorurus strongylocephalus B* (large excavators), *Hemitaurichthys zoster*, *Paracanthurus hepatus* (planktivores), *Lutjanus bohar* (piscivore), *Lutjanus fulvus*, *Lutjanus gibbus*, *Lutjanus kasmira*, *Lethrinus enigmatus* (omnivores), *Scarus niger* (scraper), *Sufflamen* spp. (invertivore) (Table 3, S2 Table, Fig 6). Note that none of these species were from the significant trophic groups detected in the permutation tests except for *Paracanthurus hepatus*. When the ordination was restricted sequentially, it showed that *Scarus niger* alone was highly correlated (rho = 0.569) with species assemblage differences. Further, a combination of only 6 species achieved a very high correlation (rho = 0.802) with species assemblage differences. Although the 13 species illustrated in Fig 6 are the best fit, other species consistently appeared in highly correlated subsets (Table 3), and therefore were likely to drive differences between fish assemblages across the archipelago. These included *Acanthurus thompsoni* (planktivore), *A*. *tennenti*, *A*. *xanthopterus* (grazer-detritivores), *Scarus frenatus* (scraper), the invertivores *Chaetodon madagascariensis* and *Sufflamen* spp. and *Lethrinus microdon* (omnivore).

**Table 3:** Species strongly correlated with differences in density of fish species assemblages across the Chagos Archipelago, based on a species level ordination (BIO-BIO) of 110 species.

Number of Species in subset	Fish Species	Spearman's Rank Correlation (rho)
**1**	*Scarus niger*	0.569
**2**	*Acanthurus thompsoni*, *Scarus niger*	0.715
**3**	*Acanthurus tennenti*, *Acanthurus thompsoni*, *Scarus niger*	0.762
**4**	*Acanthurus thompsoni*, *Naso hexacanthus S*, *Scarus niger*, *S*.*russelli*	0.767
**5**	*Acanthurus leucosternon*, *Cephalopholis sexmaculata*, *Lethrinus obsoletus*, *Scarus niger*, *Scarus psittacus*	0.783
**6**	*Acanthurus thompsoni*, *Chaetodon madagascariensis*, *Lethrinus obsoletus*, *Scarus niger*, *Sufflamen spp*., *Zebrasoma desjardinii*	0.802
**7**	*Acanthurus thompsoni*, *Chaetodon madagascariensis*, *Lethrinus obsoletus*, *N*. *hexacanthus S*, *Scarus niger*, *Sufflamen spp*., *Zebrasoma desjardinii*	0.815
**8**	*Acanthurus tennenti*, *Acanthurus xanthopterus*, *Chaetodon madagascariensis*, *Lutjanus bohar*, *Scarus frenatus*, *Scarus niger*, *Sufflamen spp*., *Zebrasoma desjardinii*	0.813
**9**	*Acanthurus tennenti*, *Acanthurus xanthopterus*, *Chaetodon madagascariensis*, *Lutjanus bohar*, *Lethrinus microdon*, *Scarus frenatus*, *Scarus niger*, *Sufflamen spp*., *Zebrasoma desjardinii*	0.818
**10**	*Acanthurus tennenti*, *Acanthurus xanthopterus*, *Canthigaster bennetti*, *Chaetodon madagascariensis*, *Lutjanus bohar*, *Lethrinus microdon*, *Scarus frenatus*, *Scarus niger*, *Sufflamen sp*., *Zebrasoma desjardinii*	0.821
**11**	*Acanthurus tennenti*, *Acanthurus xanthopterus*, *Canthigaster bennetti*, *Chaetodon madagascariensis*, *Lutjanus bohar*, *Lethrinus microdon*, *Odonus niger*, *Scarus frenatus*, *Scarus niger*, *Sufflamen spp*., *Zebrasoma desjardinii*	0.823
**12**	*Acanthurus tennenti*, *Acanthurus xanthopterus*, *Canthigaster bennetti*, *Chaetodon madagascariensis*, *Lutjanus bohar*, *Lethrinus microdon*, *Odonus niger*, *Paracanthurus hepatus*, *Scarus frenatus*, *Scarus niger*, *Sufflamen spp*., *Zebrasoma desjardinii*	0.820
**13**	*Acanthurus lineatus*, *Acanthurus nigrofuscus*, *Cetoscarus ocellatus*, *Chlorurus strongylocephalus B*, *Hemitaurichthys zoster*, *Lutjanus bohar*, *Lutjanus fulvus*, *Lutjanus gibbus*, *Lutjanus kasmira*, *Lethrinus enigmatus*, *Paracanthurus hepatus*, *Scarus niger*, *Zebrasoma desjardinii*	0.832
**14**	*Acanthurus lineatus*, *Acanthurus nigrofuscus*, *Cetoscarus ocellatus*, *Chlorurus strongylocephalus B*, *Chaetodon striatus*, *Hemitaurichthys zoster*, *Lutjanus bohar*, *Lutjanus fulvus*, *Lutjanus gibbus*, *Lutjanus kasmira*, *Lethrinus enigmatus*, *Paracanthurus hepatus*, *Scarus niger*, *Zebrasoma desjardinii*	0.824

Three broad types of fish assemblages in the Chagos Archipelago are suggested through a combination of highly significant species within the ordination (Fig 6), significant benthic associations (Fig 5) and clustering of fish species (Fig 2b). These can be defined as those aligned with: 1) higher hard coral cover (27–43%), or recently dead coral; 2) high rugosity and *Acropora* cover; and 3) higher soft coral, CCA, and macro-algal cover but low cover of live hard coral (12–22%; Table 4). The former (groups 1 and 2, Table 4) were found across Salomon and Peros Banhos atolls, whereas the latter (group 3, Table 4) was largely at GCB. It is noteworthy that two of the largest excavating parrotfishes, *Cetoscarus ocellatus* and *Chlorurus strongylocephalos* (B), showed opposing patterns of correlation (Fig 6). *Cetoscarus ocellatus* was also closely associated with *Scarus niger* on certain reefs in Salomon and Peros Banhos and both these species characterise group 1 and 2 assemblage types (Table 4). Fish assemblages at Diego Garcia Atoll forereef site were significantly different and may represent a fourth assemblage type, but there were too few survey sites to assess this. Note that 3 species were rare, present only at 1 reef (*A*. *lineatus*, *Lethrinus enigmatus* and *Lutjanus fulvus*, Table 4, S3 Table).

**Table 4:** Synthesis of results from Figs 2b, 5 and 6 and S3 and S4 Tables, to define three broad types of fish assemblages across the Chagos Archipelago, the sites at which they were found and the corresponding reef benthic characteristics. Species and benthos listed are the highest abundance/cover and were significant within analyses. Diego Garcia Atoll forereef was an outlier and is not included.

No.	Fish Species	Reefs	Benthos
1			
	*Scarus niger* (Scraper)	**Salomon**	Hard coral
	*Acanthurus nigrofuscus* (Grazer)	- terrace & forereef	*Acropora*
	*Hemitaurichthys zoster* (Planktivore)	(2 sites)	Soft coral
	*Cetoscarus ocellatus* (Excavator)	**Blenheim**	Rugosity
2			
	*Scarus niger* (Scraper)	**Peros Banhos**	Dead coral
	*Lutjanus kasmira* (Omnivore)	- lagoon (2 sites)	Rugosity
	*Cetoscarus ocellatus* (Excavator)	**Salomon**	Turf algae
	*A*. *lineatus*^a^ (Grazer)	- lagoon	Hard coral
	*L*. *gibbus* (Omnivore)	**Diego Garcia**	*Acropora*
	*Z*. *desjardinii* (Grazer)	- lagoon	
3			
	*Chlor*. *strongylocephalos* (Excavator)	**GCB**	Soft coral
	*Paracanthurus hepatus* (Planktivore)	- forereef	CCA
	*Lethrinus enigmatus*^a^ (Omnivore)	**Peros Banhos**	Macro-algae
	*Lutjanus bohar* (Piscivore)	- forereef	
	*Lutjanus fulvus*^a^ (Omnivore)	- lagoon	

^a^ rare species seen only at 1 reef.

### Species of conservation and fisheries interest

The widespread Indo-Pacific blacksaddled coralgrouper *Plectropomus laevis* was abundant and observed at all but 3 sites, with a mean density and biomass of 17.85 ± 1.54 SD individuals/ha and 104.8 ± 170.5 SD kg/ha, including several very large individuals (91–110 cm TL), close to maximum size for this species. *Plectropomus punctatus*, the marbled coralgrouper, endemic to the Indian Ocean, was never observed, yet it was recorded from the Chagos Archipelago in the 1990s by Winterbottom and Anderson [51]. No siganids were observed during the current survey, though *Siganus argenteus* and *S*. *canaliculatus* are known from the archipelago [51]. The abundance of the larger species of grouper such as *Epinephelus fuscoguttatus*, *E*. *malabaricus*, *E*. *multinotatus* and *E*. *tauvina* was extremely low, ranging from a mean of 0.0–0.77 ±1.54 SD fish/ha.

## Discussion

Large regional-scale [6,7] or long temporal-scale analyses [2] in the Indian Ocean and across the Indo-Pacific have shown that fishing and climate change are primary drivers of fish assemblage structure. We found significant differences in fish assemblage structure among the atolls of the Chagos Archipelago which we attribute to natural environmental drivers and climate change, as reflected in the significant correlations between fish assemblages and reef benthic composition. However, temporal changes before and after coral bleaching events remain unknown; future work on this would greatly enhance interpretation of the results of the current study. We can, however, assume that fishing effects are minimal due to the lack of resident human populations on any of the atolls since the 1960s (with the exception of Diego Garcia) and because of the establishment of a no-take MPA in 2010. Indeed, the Chagos Archipelago is used as a benchmark for largely unfished reefs in the Indian Ocean [7,23]. Further, our study assumed that reef fish species distributions did not differ biogeographically because of the relatively small geographic range of the Chagos Archipelago fed by the easterly flowing East African Coastal Current and South Equatorial Countercurrent, both emanating from the east African mainland [28,30], and the connectivity of the pelagic larvae of most reef fishes [29]. Of the 110 species in the dataset, there was no apparent disjunct in their distribution between the northern atolls (Peros Banhos, Salomon and Blenheim) and the southern atolls (GCB and Diego Garcia) except for *Acanthurus tristis*, *Chaetodon madagascariensis* and *Chlorurus capistratoides*, which were only found in the south, and *Chaetodon lunula* which was only found in the north. Of these, only *C*. *madagascariensis* was a significant species in the ordination analysis.

### Patterns in fish species and benthic communities

Differences in fish assemblages were significantly correlated with geographic distance between sites; the relative density of the 110 species across the archipelago differed most significantly between atolls. These atoll-scale differences were also apparent in total density and biomass values (12 families), with the highest fish densities recorded on the reefs of the western edge of the GCB, the highest fish biomass recorded at Peros Banhos Atoll and the lowest fish biomass at Diego Garcia Atoll. Reef benthic composition also varied between atolls, most notably in the relative cover of live hard coral, recently dead standing coral and rugosity, and permutation testing showed that these differences were significantly related to fish density (hard and dead standing coral) and fish biomass (hard coral, dead standing coral, rugosity). These results are not surprising since strong positive correlations between fish density or biomass and live hard coral and rugosity, benthic variables that co-vary and reflect reef habitat structural complexity, are widely reported [15,16,18, 52–54]. Therefore, patterns in the fish assemblages reported here likely reflect bottom-up control.

A major alteration in the benthic composition of coral reefs across the Indian Ocean occurred following the severe coral bleaching event of 1998 [55]. This thermal anomaly resulted in a reduction of living coral cover in the Chagos Archipelago from 50–75% cover prior to the event to ~10% live coral remaining on all six atolls in 1999 [22]. However, a majority of reef sites across the archipelago recovered rapidly and reached pre-bleaching condition by 2010 [26]. The strong benthic differences between atolls observed in our surveys in 2014 possibly reflect different levels of bleaching and differing recovery patterns following the 1998 event, though with little historic data this remains unknown. However, early reports of highly homogenous fish assemblages across reefs in the northern atolls prior to 1998 [33] suggest that the differences in the structure of the fish assemblages found in 2014 may be recent and could therefore be due to differing recovery patterns.

### Fishing effects

This study was not designed to look at fishing effects because it was based on the premise that there is no reef fishing in Chagos Archipelago, however, there was a small Mauritian fishery targeting grouper (Epinephelidae) and snapper (Lutjanidae), which operated from the 1970s until 2010 when the Chagos MPA was designated [23]. Populations of piscivore and omnivore trophic categories were similar between atolls, exemplified by the coralgrouper *Plectropomus laevis*, which was abundant and observed at all but three sites. However, two snappers *Lutjanus bohar* (piscivore) and *Lutjanus gibbus* (omnivore) were significantly correlated with differences in fish assemblages across atolls. The highest biomass of these two species was found in Peros Banhos lagoon sites (up to 861 kg/ha and 530 kg/ha, for *L*. *bohar* and *L*. *gibbus*, respectively). Apparently, the fishery did not operate in the lagoons (BIOT Fisheries Officer, pers. comm. 2014), but since our study is the first to report on fish biomass in the lagoons there are no previous comparable data. For *L*. *bohar* moderate biomass levels were found on forereefs at Salomon, Peros Banhos and Blenheim, but were lower at Diego Garcia and GCB, while biomass of *L*. *gibbus* was highly variable across all forereefs. Our surveys also suggest that three large species of grouper, *Epinephelus fuscoguttatus*, *E*. *multinotatus* and *E*. *tauvina* may have been overfished in the past since they were absent at most sites. While there is also some illegal fishing in BIOT, 80% by weight of illegal catches detected by the BIOT patrol vessel is shark [56] and therefore this poaching can be considered minimal in terms of impacts on reef fishes. Our results suggest that further research is needed to distinguish between possible latent fishing effects or natural biotic/abiotic drivers of some grouper and *Lutjanus bohar*.

A recreational fishery operates outside the MPA at the naval base in Diego Garcia and is having an impact on fish biomass [23]. Our total biomass estimates with maximum values of ~3,500 kg/ha (12 families) do not include sharks and trevally and therefore cannot be directly compared with the estimates of >9,000 kg/ha reported from 2010–2012. However, comparing relative biomass between atolls from the 2010–2012 survey [23] with our survey in 2014 shows similar differences, with highest values at Peros Banhos, followed by GCB, then Salomon, and the lowest values at Diego Garcia. This supports Graham et al.’s [23] conclusions that the recreational fishery is having an impact. Nevertheless, we measured extremely high biomass values at 10 of the 13 sites (1,501–3,000 kg/ha at six sites, and > 3,000 kg/ha at four sites). These biomass estimates are similar, when the same families are considered, to biomass at other uninhabited and protected reefs of the French territories in the Mozambique Channel [57], providing strong support for using Chagos Archipelago as a reference benchmark for unfished reef fish populations in the WIO.

### Trophic dynamics in the reef fish assemblages

Herbivory and detritivory contribute significantly to the trophic dynamics and hence biomass production on coral reefs [58,59]. Indeed, the diversity of herbivores and detritivores seen on modern reefs, illustrated by the parrotfishes (Labridae: Scarinae) and surgeonfishes (Acanthuridae), has been linked to the massive expansion of shallow coral reef habitats over the last 5 million years [60]. In the Chagos Archipelago, the grazer-detritivores was the trophic group that differed most significantly between atolls. This group comprises a suite of acanthurids (“ring-tail” surgeonfishes [44]), such as *Acanthurus tennenti* and *A*. *xanthopterus*, that harvest mouthfuls of soft sediment on dead coral substrate, as well as on sand, which contain the diatoms and microbes of their diet [61,62]. Their highest densities at GCB and Diego Garcia (>500 and >600 individuals/ha, respectively) corresponded with low hard coral cover. In contrast, low numbers of these surgeonfishes were seen at Peros Banhos, Salomon and Blenheim (<130, <14, <170 individuals/ha, respectively), where hard coral cover was high. These results suggest that these “grazer-detritivore” surgeonfish species may thrive where their benthic food sources have increased due to coral mortality [63] so potentially may serve as indicators of reef degradation. The prevalence of the detritivory role is also supported by one of the most common reef fishes in the world, the bristletooth surgeonfishes *Ctenochaetus* spp. [64], with the combined density of two species *Ctenochaetus truncatus* and *C*. *striatus* of ~850 individuals/ha, the second highest of all the 110 species surveyed (*Caesio* spp. density was the highest: 936 individuals/ha). We propose that the importance of detritivory in recovery of degraded reefs and in cycling carbon within coral reef systems is not well quantified and therefore an important area for future research.

A strong relationship between hard coral cover and corallivores has been widely reported [23,54,65] and was confirmed here with significantly higher densities of obligate coral-feeding butterflyfishes at Peros Banhos and Salomon atolls where there was relatively higher live coral cover. These coral specialists are clearly highly vulnerable to coral mortality and, as such, have long been used as potential indicator species for monitoring coral reef health [66]. The third trophic group that differed significantly between atolls was the planktivores, comprising several acanthurids (three *Naso* spp., *Acanthurus thompsoni* and *Paracanthurus hepatus)*, two chaetodontids, two balistids and *Caesio* spp. The biomass of this group was three times higher at GCB, with a mean biomass of 1,045 kg/ha, compared to 338kg/ha for other atolls, and this was largely due to the caesionids and *Naso hexacanthus* and *N*. *brevirostris*. Further, three planktivores were strongly correlated with the ordination: *Paracanthurus hepatus*, *Acanthurus thompsoni* and the chaetodon *Hemitaurichthys zoster*. Drivers of planktivore populations on coral reefs are still poorly understood, but their food items are associated with reef edges and proximity to deep water [67]. These acanthurid species are all zooplanktivores [68], suggesting waters at GCB may be zooplankton-rich. Thus, higher Acanthuridae densities overall at GCB reefs appear to reflect two different and unrelated trophic pathways: increased access to soft benthic surfaces due to coral mortality for the grazer-detritivores and higher zooplankton densities for planktivores. Finally, it was notable that the density and biomass of the piscivore and omnivore trophic groups, species that represent important target fishery species [23,39], were not significantly correlated with fish assemblage patterns across the archipelago, suggesting that benthic differences did not directly affect these higher trophic level taxa. Thus, when data were aggregated by trophic group, only three groups differed significantly and these appeared to be influenced by reduced coral cover due to climate change [22] and natural variability in zooplankton, both bottom-up control pathways.

It was surprising that none of the five herbivore trophic groups, which included all the parrotfishes, were significant in explaining differences in fish assemblages between sites. We found species-level analyses were more informative than aggregated trophic group analyses and demonstrated species from within seven trophic groups were highly related to differences in the fish assemblages across the reefs of the Chagos Archipelago. *Scarus niger* had the strongest correlation with fish assemblage structure across the archipelago, with highest biomass on the high coral cover northern atolls (182 kg/ha at Peros Banhos), and the lowest at Diego Garcia and the low coral cover reefs of GCB (3 kg/ha and 11–27 kg/ha, respectively). This species is one of the most ubiquitous parrotfishes across the Indo-Pacific [69,70] and feeds on the top 1–2 mm of dead coral substrate [62,71], though it probably removes epilithic algae while feeding. *Scarus niger* was associated with the highly abundant surgeonfish *Acanthurus nigrofuscus*, known to graze similar substrate types but feeding on epilithic algae [60,62]. These two species correlated most closely with the assemblages at Salomon Atoll sites, particularly on the outer forereefs where live hard coral, *Acropora* and rugosity were highest, but also at the Peros Banhos lagoon sites where turf algae and dead coral were relatively high. This result may reflect “feeding complimentarity” by a parrotfish and a surgeonfish, accessing different algal prey within the same benthic substrate [72]. It also illustrates the challenges in using trophic categories as a proxy for ecological function. Herbivorous fishes have been implicated in the top-down control of reef benthos, as their grazing of recently dead coral substrate prevents the rapid colonisation of macroalgae. Further, over-fishing of herbivores has been invoked to explain declines in coral cover and they are consequently considered to play a key functional role in maintaining coral reef resilience [4,11,12,73–75]. Parrotfishes (Labridae; Scarinae) are a significant component of this herbivorous fish community on account of their size, numerical abundance and hence biomass [14]. They are also targeted in many reef fisheries and are frequently used as indicators for the condition or resilience of reefs [14,39,44,76]. However, recent work on the intricacies of parrotfish feeding modes and diets [14,60,71] indicates that assigning species with similar feeding modes into broad trophic groups may over-simplify their functional role in reef resilience. Further, we show that parrotfish population densities can vary by up to 43-fold between reef sites in the absence of fishing and so caution against assumptions that declines in parrotfish populations are necessarily due to fishing.

The largest parrotfishes, *Cetoscarus ocellatus* and *Chlororus strongylocephalos*, showed completely opposing patterns in their distribution with *Cetoscarus ocellatus* closely associated with healthy reefs with high coral cover at the northern atolls (Peros Banhos and Salomon). In contrast, *Chlorurus strongylocephalos* was strongly correlated with reefs at GCB which had the lowest live coral and the highest cover of calcareous algae, soft coral and macroalgae. This opposing pattern in the distribution of these two high-biomass parrotfish, functionally termed “excavators” [14,44, 63], can be explained by their feeding behaviour. *Cetoscarus ocellatus* are territorial, non-schooling, harvest small areas of reef and are associated with reefs of high live coral cover (M. Samoilys, pers. obs.). In contrast, *Chlorurus strongylocephalos* prefers disturbed reefs which offer a larger benthic surface area for excavating the dead coral reef matrix [71]. They feed on these substrates, typically in large schools if the disturbed substrate is of sufficient area (H. Choat, James Cook University, pers. comm. 2016). The Indian Ocean endemic, *Chlorurus enneacanthus*, was observed to have a similar feeding strategy to *Chlorurus strongylocephalos* (M. Samoilys pers. obs.). These *Chlorurus* species conform to reports from the Philippines where some parrotfish species prefer areas of reef that have become damaged, for example from cyclones [21]. The patterns seen here suggest bottom-up control of parrotfish populations by coral cover in positive (e.g. *S*. *niger*, *C*. *ocellatus*) or negative (e.g. *C*. *strongylocephalos*) relationships. These pathways therefore need to be considered when examining the role of parrotfishes in influencing coral recovery trajectories.

### Conclusions

The isolated Chagos Archipelago provides a valuable ecological benchmark for understanding the structure of reef fish assemblages when fishing impacts are minimal. Differences in fish assemblages across the archipelago were associated with variation in reef benthic condition, which suggested a bottom-up response of fish populations to changes in coral cover. Our results support the concept that herbivory and detrivory are significant functions provided by reef fishes [58–60], but we propose that separating diet from the structural impact of these feeding modes will improve our understanding of their functional role in reef resilience. The large variation in parrotfish abundance found in the Chagos Archipelago supports studies (e.g. Russ et al. [21]) that caution against assumptions elsewhere that parrotfish population abundances are largely driven by fishing. We found surgeonfish species that graze epilithic algae and parrotfish species that exploit bare substrate to access nutrients within the calcareous matrix [61,62,71] are two key taxa responsible for differences in fish assemblages among the atolls. Both may function to keep macroalgal levels down, but the drivers of their populations are different. Parrotfishes have evolved highly successful traits to exploit food sources on reefs and contribute significant biomass on coral reefs [14, 60], including during declines in coral cover [21,63] and some species are impacted negatively by fishing [63]. Clarifying these trophic dynamics is vital to refine functional trait approaches for understanding the impacts of climate change and fishing on coral reef biodiversity.

## Supporting information

S1 Table Fish and benthos survey sites with reef type descriptors at each atoll. Two dive surveys for fish were done at each site. * = fish survey sites where no benthic data were collected.
(DOCX)

S2 Table Taxa surveyed for abundance and biomass and their trophic group and functional characteristics. All taxa were recorded to species level (not all species are listed here). Those split by body size are species that change diet with size. Trophic categories adapted from Green and Bellwood 2009, Sandin and Williams 2010, and feeding information based on Choat and Clements 1998, 2010; Clements et al. 2016. Species listed in {parentheses} are WIO species but were not observed in this study.
(DOCX)

S3 Table Mean density per hectare ± SD for each fish species per site. (XLS)

S4 Table Mean biomass (kg) per hectare ± SD for each fish species per site. (XLS)

S5 Table Mean percentage cover for each benthic category per hectare ± SD per site. (XLSX)
