# Supplementary material for: Patterns in reef fish assemblages: Insights from the Chagos Archipelago
Source: PLoS One. 2018 Jan 19;13(1):e0191448. doi: 10.1371/journal.pone.0191448 (PMC5774777; doi:10.1371/journal.pone.0191448)
Supplement: S1 Table — Two dive surveys for fish were done at each site. * = fish survey sites where no benthic data were collected. (DOCX) [file pone.0191448.s001.docx]

**Table S1.** Fish and benthos survey sites with reef type descriptors at each atoll. Two dive surveys for fish were done at each site. *= fish survey sites where no benthic data were collected.

| Atoll | Site name | Reef type | Fish transect depths |
| --- | --- | --- | --- |
| Blenheim Reef | Blenheim | Forereef | 7–15 |
| Salomon Atoll | SAIsle Anglaise | Terrace & forereef | 7–20 |
|  | SAIsdela Passe | Terrace & forereef | 4–23 |
|  | Salomon Lagoon | Lagoon pinnacle | 3–20 |
| Peros Banhos Atoll | PBIsdela Passe | Terrace & forereef | 7–16 |
|  | PBDiamantLagoon* | Lagoon inner slope | 3–7 |
|  | PBCoinLagoon | Lagoon inner slope | 3–15 |
|  | PBLagoonS | Lagoon pinnacle | 3–17 |
| Great Chagos Bank | Three Brothers | Terrace & forereef | 6–17 |
|  | EagleOut | Forereef | 10–16 |
|  | EgmontIn | Forereef | 8–17 |
| Diego Garcia Atoll | DGOuterN | Terrace & forereef | 8–16 |
|  | DGLagoonN* | Lagoon pinnacle | 9–18 |
