# Supplementary material for: Patterns in reef fish assemblages: Insights from the Chagos Archipelago
Source: PLoS One. 2018 Jan 19;13(1):e0191448. doi: 10.1371/journal.pone.0191448 (PMC5774777; doi:10.1371/journal.pone.0191448)
Supplement: S2 Table — All taxa were recorded to species level (not all species are listed here). Those split by body size are species that change diet with size. Trophic categories adapted from Green and Bellwood 2009, Sandin and Williams 2010, and feeding information based on Choat and Clements 1998, 2010; Clements et al. 2016. Species listed in {parentheses} are WIO species but were not observed in this study. (DOCX) [file pone.0191448.s002.docx]

**Table S2.** Taxa surveyed for abundance and biomass and their trophic group and functional characteristics. All taxa were recorded to species level (not all species are listed here). Those split by body size are species that change diet with size. Trophic categories adapted from Green and Bellwood 2009, Sandin and Williams 2010, and feeding information based on Choat and Clements 1998, 2010; Clements et al. 2016. Species listed in {parentheses} are WIO species but were not observed in this study.

| **Functional Group** | **Notes on feeding habits and selection of species** | **Group/family** | **English name or species** |
| --- | --- | --- | --- |
| ***Piscivores*** | top level predators, exert top-down control on lower trophic levels of fish, are vulnerable to overfishing therefore good indicators of the level of fishing on a reef. | Serranidae  Lutjanidae | All groupers  *Aprion viriscens*  *Lutjanus bohar* |
| ***Omnivores*** | Secondary-level predators/ consumers with highly mixed diets including small fish, invertebrates and dead animals. Their abundance a good indicator of fishing pressure | Haemulidae  Lethrinidae  Lutjanidae | All sweetlip  All emperor  All snapper except *Aprion viriscens & Lutjanus bohar* |
| ***Corallivores*** | Obligate and facultative corallivores are a secondary indicator of coral community health. | Chaetodontidae | 8 Butterflyfish: *C. bennetti,*  *C. lineolatus, {C. melannotus},*  *C. meyeri, {C. ornatissimus},*  *C. trifascialis, C. trifasciatus,*  *C. zanzibarensis* |
| ***Invertivores*** | Feed on coral competitors such as soft corals and sponges, their abundance may be a secondary indicator of stability of these groups and of a phase shift. Also prey on small invertebrates in the benthos. | Pomacanthidae  Balistidae | Angelfish. All species except *Centropyge* spp. which are grazer-detrivores  Benthic triggerfish (e.g. *Sufflamen* spp.) |
|  |  | Chaetodontidae | Non-corallivore Butterflyfish: all other Chaeotdontids except *H. zoster* and *H. diphreutes* which are planktivores |
| ***Planktivores*** | Resident on reefs but feed in the water column. Their presence/absence may be related to water column conditions, suitable habitat for shelter or reef features such as passes | Chaetodontidae  Balistidae | *Hemitaurichthys zoster, Heniochus diphreutes*  Triggerfish in the water column eg. *Melichthys* spp., *Odonus niger* |
|  |  | Acanthuridae | *A. mata, A. nubilus, A. thompsoni, Paracanthurus hepatus*  All large *Naso* (>20cm TL, 16cm for *N. hexacanthus*), except *N.* *unicornis, N. elegans, N.* *tuberosus, N. brachycentron* which are Browsers |
|  |  | Caesionidae | All Fusiliers |
| ***Detritivores*** | Feed on organic matter including diatoms in sediment and reef surfaces, high abundances poorly understood, may play an important role in reef repair or be linked to eutrophication | Acanthuridae | *Ctenochaetus spp.* |
| ***Grazer-detritivores*** | Feed on algal turf and sediment to extract detritus, microbes and diatoms; may limit growth of macroalgae | Acanthuridae  Pomacanthidae | *A. blochii, A. dussumieri, A. leucocheilus, A. nigricauda,*  *A. xanthopterus, A. tennenti*  *Centropyge* spp*.* |
| ***Herbivores*** | Feed on endolithic and epilithic algae, substratum and macro-algae. Exert control on coral-algal dynamics, implicated in determining phase shifts from coral to algal dominance e.g in response to mass coral mortality | | |
| *Large excavators* | Take few, large, deep bites, and remove calcareous substratum; play a large role in bioerosion | Scarinae | *Chlorurus spp. >35cm,* e.g. *C. strongylocephalos*  *Cetoscarus ocellatus*  *{Bolbometapon muricatum}* |
| *Small excavators* | Remove algae and substrate; play a smaller role in bioerosion | Scarinae | *Chlorurus spp. <35cm* |
| *Scrapers* | Remove algae, sediment and detritus by closely cropping or scraping the substrate |  | *Scarus spp., Hipposcarus spp.* |
| *Browsers* | Feed on large macro-algae | Scarinae | *Calotomus* spp.  *Leptoscarus* spp. |
|  |  | Acanthuridae | *Naso elegans, N. tuberosus, N. unicornis,N. brachycentron,* all other *Naso spp.* <21cm (<16cm for *N. hecacanthus*) |
|  |  | Ephippidae | Bat fish – *Platax* spp. |
|  |  | Kyphosidae | Rudder fish – *Kyphosus* spp. |
| *Grazers* | Graze epilithic algal turfs, including red algae; likely to limit growth of macroalgae | Acanthuridae  Siganidae | *Zebrasoma* spp.  *A. nigrofuscus,* other *Acanthurus spp.* e.g, *A. lineatus* |

Additional References

Sandin S, Williams I. Trophic classification of reef fishes from the Tropical U.S. Pacific (Version 1.0). Series: Scripps Institution of Oceanography Report. San Diego, California. 2010; 19pp.
